# Pennsylvania College of Medicine

**Published:** 1856-09

**Authors:** 


### PENNSYLVANIA COLLEGE OF MEDICINE.

We have (since the notice of other Institutions) received the announcement of the Pennsylvania Medical College, with the advertisement of the next regular Course of Lectures, (to be found upon the cover of this number,) commencing on Monday, the 13th of October, and continuing until the 1st of March. Though it has never been our pleasure to visit the Institution, from the high character of the gentlemen composing the Faculty, we are satisfied that it is one of the most promising Colleges in the Union.

